# Nonsteroidal Mineralocorticoid Receptor Antagonist (Finerenone) in Cardiorenal Disease

**DOI:** 10.3390/jcm12196285

**Published:** 2023-09-29

**Authors:** Monarch Shah, Alaa S. Awad, Emaad M Abdel-Rahman

**Affiliations:** 1 Division of Nephrology, University of Virginia, Charlottesville, VA 22902, USA; monarch.shah08@gmail.com; 2 Division of Nephrology, University of Florida, Jacksonville, FL 32209, USA; alaa.awad@jax.ufl.edu

**Keywords:** finerenone, cardiorenal disease, chronic kidney disease, mineralocorticoid receptor antagonist, heart failure

## Abstract

Nonsteroidal mineralocorticoid receptor antagonists (MRAs) present a promising therapeutic option in cardiorenal diseases, mitigating the limitations of steroidal MRAs. Finerenone, a third-generation nonsteroidal MRA, has demonstrated beneficial effects in heart failure (HF) and chronic kidney disease (CKD). Clinical trials, including FIDELIO-DKD and FIGARO-DKD, revealed finerenone’s efficacy in improving kidney and cardiovascular (CV) outcomes. Patients with CKD and type 2 diabetes (T2DM) on finerenone experienced reduced rates of cardiovascular events, including hospitalization for HF. However, these trials excluded symptomatic HF patients, focusing on asymptomatic or early-stage HF. The ongoing FINEARTS-HF trial evaluates finerenone in HF with preserved ejection fraction (HFpEF). Additionally, studies exploring finerenone and sodium-glucose cotransporter 2 (SGLT2) inhibitors’ (Empagliflozin) combination effects in CKD and T2DM (CONFIDENCE) and the selective MR modulator AZD9977 with another SGLT2 inhibitor (dapagliflozin) in HF and CKD (MIRACLE) aim to expand treatment options. While SGLT-2 inhibitors were shown to reduce hyperkalemia risk in FIDELIO-DKD and potentially lower new-onset HF incidence in FIGARO-DKD, further research is essential. So far, the evidence for the beneficial effect of finerenone in the spectrum of cardiorenal diseases is based only on the results of studies conducted in patients with T2DM, and clinical trials of finerenone in patients with nondiabetic kidney disease are ongoing. Nonsteroidal MRAs hold significant potential as pivotal treatment targets across the cardiorenal disease spectrum. This review will focus on the effects of finerenone on cardiorenal disease.

## 1. Introduction

Steroidal mineralocorticoid receptor (MR) antagonists (MRAs), namely spironolactone and eplerenone, elicit antihypertensive effects and ameliorate the progression of chronic kidney disease (CKD). Nevertheless, their clinical application is limited by their side effects, including hyperkalemia, gynecomastia, impotence, and amenorrhea [1]. Therefore, recent advancements aimed to develop nonsteroidal MRAs with a potent affinity for the mineralocorticoid receptor to mitigate the undesired adverse effects of steroidal MRAs. In 2021, the Food and Drug Administration (FDA) approved finerenone, marking the introduction of the first nonsteroidal mineralocorticoid antagonist (MRA) to the market. Currently, nonsteroidal MRAs are undergoing assessment for their efficacy in HF and their potential synergistic utilization alongside sodium-glucose cotransporter 2 (SGLT2) inhibitors. These groundbreaking agents hold promise as a significant therapeutic option across the cardiorenal disease spectrum. This review aims to comprehensively outline the mechanisms and clinical assessment of nonsteroidal MRAs, while also providing insights into the current and prospects in the management of cardiorenal disease.

## 2. Mechanism of Action and Pharmacodynamics of Nonsteroidal Mineralocorticoid Receptor Antagonists

Spironolactone was the first MRA to undergo investigation for use in HF [2,3]. Eplerenone, a second-generation mineralocorticoid receptor antagonist, stood out in comparison to spironolactone as it had reduced affinity for androgen, progesterone, and glucocorticoid receptors and a lower likelihood of related adverse side effects [4]. Both spironolactone and eplerenone are currently in use for HF with reduced ejection fraction (HFrEF). Due to their steroid-based nature, they bear a structural resemblance to aldosterone and cortisol. Consequently, a multitude of side effects associated with steroidal MRAs emerge, posing limitations or reduced desirability for their use [5]. The MRs exhibit widespread distribution across various tissues and organs, including the heart, kidney, brain, lung, colon, skin, liver, skeletal muscle, saliva, sweat glands, and adipose tissue [6]. MRs play a pivotal role in ventricular remodeling and the progression of chronic HF [7]. Finerenone, classified as a third-generation remarkably selective MRA, exerts the direct and specific inhibition of mineralocorticoid receptor hyperactivation, thereby facilitating anti-inflammatory and antifibrotic effects [6,7]. Due to its balanced distribution in the heart and kidney, finerenone exhibits cardiorenal benefits, as highlighted by Kolkhof and colleagues [8]. Finerenone elicits changes that influence the recruitment of co-activators and corepressors, consequently altering its stability, nuclear translocation, and activity. Phase IIa clinical trials conducted by Ruilope et al. and Bakris et al. [9,10] involved the administration of finerenone to patients with T2DM (T2DM) and HF, accompanied by renal dysfunction. Finerenone demonstrates a level of effectiveness in blocking the mineralocorticoid receptor similar to spironolactone. Moreover, its lower lipophilicity and higher polarity result in restricted blood–brain barrier penetration and uniform distribution within the heart and kidney tissues, as elucidated by Pandey et al. [11]. Following oral administration, finerenone is completely absorbed through the gastrointestinal tract. The primary route of metabolism occurs predominantly via the cytochrome CYP3A4 enzyme, accounting for 90% of its metabolic transformation into an inactive metabolite. Subsequently, approximately 80% of finerenone is excreted in the urine, while the remaining portion is eliminated through fecal excretion [12]. Importantly, unlike spironolactone and eplerenone, finerenone has a short half-life that may explain its minimal effects on serum potassium levels [13].

## 3. Cardiorenal Disease Spectrum

Aldosterone predominantly exerts its effects in the distal tubule of the nephron, promoting the enhanced reabsorption of water and sodium, which in turn leads to extracellular fluid expansion. Notably, extensive research has compellingly demonstrated that beyond its regulatory role in body fluid and electrolyte balance, aldosterone and its interaction with the MRs profoundly impacts diverse cellular functions, culminating in inflammation and tissue fibrosis. Furthermore, it is noteworthy that aldosterone production may also occur locally in peripheral tissues [14]. Evidence suggests that prolonged exposure to inappropriately elevated levels of aldosterone results in cardiac and renal damage, irrespective of blood pressure levels [15,16], by promoting renal fibrosis, inflammation, and cardiac stiffness. The MR is subject to activation by both aldosterone and cortisol [11]. In epithelial cells, particularly in the renal tubules, aldosterone is presumed to function as the principal ligand. Upon aldosterone activation in renal cells, there is a cascading effect of sodium and fluid retention, accompanied by potassium excretion [17]. Conversely, in nonepithelial cells like myeloid cells and macrophages, the mineralocorticoid receptor responds to both aldosterone and cortisol as activating ligands. Prolonged overactivation by these two ligands in such cells contributes to fibrosis, inflammation, and remodeling processes in the heart, kidney, and blood vessels [11]. The kidneys play a central role in the long-term regulation of arterial pressure, accomplished through the homeostatic control of sodium balance and the maintenance of extracellular fluid volume via the renin–angiotensin–aldosterone system (RAAS) [18,19]. Aldosterone directly influences sodium retention and, consequently, has a direct impact on blood pressure regulation [20]. Overactivation of the MR in the kidney results in glomerular hypertrophy, sclerosis, and renal fibrosis, ultimately causing renal dysfunction [21]. Low-grade inflammation is a characteristic feature of the cardiorenal disease spectrum. Patients with hypertension, CV disease, and renal dysfunction frequently exhibit chronic vascular inflammation. In adult rat models, mineralocorticoid receptor overactivation heightens NADPH oxidase activity, prompting a sequence of oxidative stress responses. This cascade leads to the inflammatory and fibrotic processes, ultimately resulting in cardiac lesions, including myocardial hypertrophy and ventricular remodeling, leading to the progression of CV and renal diseases [22,23]. The risks of CV events and new-onset HF escalate when the urinary albumin-to-creatinine ratio (UACR) surpasses 10 and the estimated glomerular filtration rate (eGFR) declines below 75 mL per minute per 1.73 m^2^ of body surface area [24]. MR overactivation is recognized as a significant contributor to the pathophysiology of cardiorenal syndrome, making it a pivotal therapeutic target [8].

## 4. Current Evidence in Patients with Heart Failure and Chronic Kidney Disease

Many classes of drugs are used in medical therapy for HF such as diuretics, beta blockers, RAAS blockade, SGLT2 inhibitor, nitrates and MRAs, many of which are also used for the prevention of CKD progression. Diabetes is the most common cause of CKD in the USA. Despite guideline-directed therapy, the risk of renal failure is high in diabetics. The renoprotective effect of RAAS blockers is well known, but 40% of patients experience “aldosterone escape” where serum aldosterone rises to pretreatment levels after many weeks of RAAS blockade [25]. SGLT2 inhibition reduces glucose and sodium reabsorption in the proximal tubule, thereby increasing the delivery of sodium to the macula densa, causing afferent arteriolar vasoconstriction. Thus, intraglomerular pressure decreases, reducing albuminuria as well [26]. Rat models have shown that dapagliflozin administration reduced RAAS activation by downregulation of the angiotensin II type 2 receptor expression in the kidneys [27]. Overall, SGLT2 inhibition influences renal hemodynamics, blood pressure, and proteinuria, thus providing benefit when added to RAAS blockade. Given RAAS upregulation and MR-induced and CV inflammation and remodeling, steroidal MRAs like spironolactone and eplerenone have had an important role in treatment.

In 2021, finerenone received FDA approval for use in patients with CKD associated with T2DM. Early indications of finerenone’s beneficial impact on cardiac function in HF were observed in the phase II Mineralocorticoid Receptor Antagonist Tolerability Study (ARTS) trial (Table 1), a safety and tolerability study. This randomized controlled trial compared the safety and tolerability of finerenone at daily doses ranging from 2.5 to 10 mg with spironolactone at 25 or 50 mg daily in patients diagnosed with New York Heart Association (NYHA) class II-III HF, exhibiting reduced ejection fraction (≤40%) and mild or moderate CKD (eGFR range of 30–90 mL/min/1.73 m^2^) over 30 days [28]. Notably, finerenone led to a greater reduction in systolic blood pressure and exhibited significantly lower incidences of hyperkalemia and less decline in eGFR, while the albuminuria remained unaffected compared to spironolactone across all doses [28]. Overall, finerenone at doses of 5–10 mg/day was found to be equally effective to spironolactone at doses of 25–50 mg/day in the study [28].

The ARTS-HF trial was a randomized double-blind study that compared the effects of finerenone (at doses up to 20 mg/day) and eplerenone (at doses ranging from 25 mg/day to 50 mg/day) in patients diagnosed with both T2DM and CKD, who were experiencing worsening HFrEF within 7 days of hospitalization. Patients treated with finerenone demonstrated good tolerability, and a comparable percentage of patients on eplerenone experienced a reduction of 30% or more in N-terminal prohormone of brain natriuretic peptide (NT-proBNP) levels. The safety profile of finerenone was similar to that of eplerenone [29].

A meta-analysis of the ARTS, ARTS- HF, and ARTS- HF Japan trials by Pei et al. [30] showed that finerenone demonstrated a dose-dependent reduction in NT-proBNP levels, UACR, and other relevant biochemical markers. Regarding its efficacy in anti-ventricular remodeling among patients with chronic HF, finerenone at a 10 mg/day dosage was found to be as effective as steroidal MRAs at doses ranging from 20 to 50 mg/day [30].

The successful outcomes observed in the ARTS series paved the way for two groundbreaking phase III trials: “Finerenone in reducing kidney failure and disease progression in diabetic kidney disease” (FIDELIO-DKD) and “Finerenone in Reducing Cardiovascular Mortality and Morbidity in Diabetic Kidney Disease” (FIGARO-DKD). In the FIDELIO-DKD trial, patients were randomized in a double-blind manner to receive finerenone at daily doses of 10 or 20 mg or a placebo. The study enrolled patients with either a UACR of 30–300 mg/g and an eGFR of 25–60 mL/min/1.73 m^2^ with diabetic retinopathy or a UACR of 300–5000 mg/g and an eGFR of 25–75 mL/min/1.73 m^2^ [31].

The FIDELIO-DKD trial was pivotal in obtaining FDA approval for finerenone and provided essential insights into the appropriate patient population for its use and the expected benefit-to-risk ratio. The study demonstrated that finerenone improved kidney outcomes in patients with predominantly stage 3 or 4 CKD and severely elevated albuminuria, combined with T2DM, a population at high risk for kidney-related complications [31]. Additionally, as a secondary outcome, the trial assessed a composite CV endpoint consisting of time to CV death, myocardial infarction, stroke, or HF hospitalization. Patients treated with finerenone showed significantly decreased rates of CV events (95% CI 0.75–0.99; *p* = 0.03) compared to those on placebo [32], mainly via reducing HF hospitalization rate.

In the FIGARO-DKD trial, despite excluding patients with symptomatic HF and reduced ejection fraction, hospitalization for HF emerged as a significant driver of the primary outcome [33]. Considering that patients with CKD and T2DM, along with new-onset or preexisting HF, face major risks of hospitalization and mortality, finerenone treatment offers a promising advancement in HF prevention and management, thereby reducing substantial healthcare burdens in such patient populations. Although the effects of finerenone treatment on the kidney composite outcome (including a decrease of at least 40% in eGFR from baseline) were comparable in the FIGARO-DKD and FIDELIO-DKD trials, statistical significance was not attained. However, the incidence of end-stage kidney disease was found to be lower in the finerenone group compared to the placebo group. 

In both trials, the occurrence of the composite kidney outcome (reduction in the eGFR of at least 57% from baseline (more sensitive compared to an eGFR decrease of ≥40%) was lower in the finerenone group [33]. Patients with symptomatic HFrEF classified as NYHA class II-IV were excluded from both the FIGARO-DKD and FIDELIO-DKD trials. Consequently, participants with HF at baseline fell into the following categories: those with asymptomatic HFrEF, HFrEF with NYHA class I, HF with mid-range ejection fraction (HFmrEF), or HF with preserved ejection fraction (HFpEF) [34,35]. Finerenone demonstrated a significant reduction in the incidence of new-onset HF when compared to the placebo [34]. In the FIDELIO-DKD analysis, a total of 436 participants, representing 7.7% of the overall population, had a history of HF [35].

A prespecified pooled analysis, known as FIDELITY, was conducted on data from both phase 3 trials (Combined FIDELIO-DKD and FIGA-RO-DKD Trial Programme Analysis) that demonstrated that finerenone treatment had beneficial effects on both CV and kidney outcomes, showing improvements across the various stages of CKD severity [36,37].

Steroidal MRAs have been proven to be advantageous in CKD by effectively mitigating endothelial dysfunction and oxidative stress, thereby leading to a reduction in the degree of albuminuria [38].

Finerenone exhibited a comparable impact on nephropathy, resulting in a reduction in albuminuria and an improvement in eGFR. These beneficial effects were attributed to increased nitric oxide concentrations and reduced oxidative stress [39,40].

A recent study conducted on ZSF1 rats, an established preclinical model of HFpEF in male hypertensive and diabetic rats, revealed that finerenone treatment (at a dose of 10 mg/kg/day for 12 weeks) resulted in reduced kidney hypertrophy and cardiac fibrosis, while also improving cardiac diastolic function and perfusion [41]. Finerenone demonstrated a significant amelioration of diastolic dysfunction and enhanced cardiac perfusion in ZSF1 rats. Interestingly, these cardiac benefits were largely independent of the renal benefits observed [41].

**Table 1:** Summary of current evidence on finerenone in patients with heart failure and chronic kidney disease.

Studies	Duration	Sample Size	Study Design	Inclusion Criteria	Primary Outcome	Secondary Outcome	Conclusion
ARTS (Part A: finerenone vs. placebo; part B: finerenone vs. spironolactone or placebo) 2012 [28]	30 days	458	Multicenter, phase II study, with double-blind placebo and open-label spironolactone	HFrEF (NYHA II-III, LVEF ≤ 40%) Mild or moderate CKD (eGFR 60 to <90 (Part A) and 30–60 mL/min/1.73 m^2^ (Part B))	Change in the serum potassium concentration vs. placebo	Changes in the serum potassium concentration vs. spironolactone Changes in the biomarkers of cardiac and kidney function or injury, eGFR (MDRD), and albuminuria	Significantly smaller increases in potassium levels with finerenone than with spironolactone. Finerenone was at least as effective as spironolactone in lowering NT-proBNP levels and albuminuria.
ARTS—HF (finerenone vs. eplerenone) 2016 [29]	90 days	1066	Randomized, double-blind, phase 2b multicenter study	Worsening HF with HFrEF exacerbation and CKD and/or T2DM requiring hospitalization and intravenous diuretic therapy	Percent of patients with decrease of >30% of NT pro-BNP until day 90.	All-cause death, CV hospitalizations, or worsening HF occurred least frequently in the finerenone 10 mg group versus the eplerenone group (hazard ratio 0.56; *p* = 0.016). All-cause death (*p* = 0.062) and CV death (*p* = 0.011) occurred less frequently in the finerenone versus eplerenone groups.	Finerenone is well tolerated and led to a ≥30% decrease in NT-proBNP levels similar to eplerenone. All-cause death, CV hospitalization, or acute worsening HF was less common with finerenone than with eplerenone.
ARTS—DN (finerenone vs. placebo) 2015 [14]	90 days	823	Multicenter, double-blind, placebo controlled, phase II RCT	CKD and T2DM receiving renin–angiotensin system inhibitors. UACR ≥ 30 mg/g and eGFR > 30 mL/min/1.73 m^2^	Change in UACR over 90 days vs. at baseline.	Hyperkalemia leading to study drug discontinuation was 2.1% in the 7.5 mg group, 0% in the 10 mg group, 3.2% in the 15 mg group, 1.7% in the 20 mg group, and 1.5% for placebo.	UACR reduction was dose-dependent in the finerenone group compared to placebo, addition of finerenone compared with placebo resulted in improvement in the UACR. Permanent drug discontinuation due to hyperkalemia not seen with placebo or finerenone 10 mg/day.
FIDELIO-DKD (finerenone vs. placebo) 2020 [31]	2.6 years	5674	Randomized, double-blind, placebo controlled, phase 3 multicenter study	T2DM Predominantly advanced CKD (on maximal dose of ACEi/ARB) with severely increased albuminuria	Time to onset of kidney failure, time to sustained eGFR decrease of at least 40% from baseline over at least 4 weeks or death from a renal cause.	CV death, myocardial infarction (MI), stroke, hospitalization for HF (HHF): 13% vs. 14.8% (*p* = 0.03) CV death: 4.5% vs. 5.3% (*p* > 0.05) Nonfatal MI: 2.5% vs. 3.1% HHF: 4.9% vs. 5.7% Hyperkalemia: 15.8% vs. 7.8%	In patients with CKD and T2DM, finerenone lowers the risks of CKD progression and CV events compared to the placebo.
FIGARO-DKD (finerenone vs. placebo) [33]	3.4 years	7352	Randomized, double-blind, placebo controlled, phase 3 multicenter study	T2DM CKD (Stage 2–4 CKD with moderately increased albuminuria or stage 1–2 CKD with severely increased albuminuria)	CV death and non-fatal CV events (i.e., MI, stroke, hospitalization for HF).	Composite of the first occurrence of kidney failure (sustained decrease from baseline of ≥40% in GFR, or death from renal cause): 9.5% vs. 10.8% (HR 0.87, 95% CI 0.76–1.01) End-stage kidney disease: 0.9% vs. 1.3% (HR 0.64, 95% CI 0.41–0.995) All-cause hospitalizations: 42.7% vs. 43.8% All-cause mortality: 9% vs. 10.1% (HR 0.89, 95% CI 0.77–1.04) Hyperkalemia: 10.8% vs. 5.3%	Finerenone therapy improved CV outcomes as compared with placebo.

Abbreviations: HFrEF: Heart failure with reduced ejection fraction, NYHA: New York Heart Association, CKD: Chronic kidney disease, eGFR: Estimated glomerular filtration rate, MDRD: Modification of Diet in Renal Disease, LVEF: Left ventricular ejection fraction, NT pro-BNP: N-terminal pro-brain natriuretic peptide, T2DM: Type 2 diabetes mellitus, CV: Cardiovascular, RCT: Randomized control trial, UACR: Urine albumin creatinine ratio, HHF: Hospitalization for heart failure, MI: Myocardial infarction, CI: Confidence interval; HR: Hazard ratio.

## 5. Future Directions

CV and kidney disease outcomes associated with finerenone have already been extensively studied in patients with T2DM and CKD. The ongoing FIND-CKD study [42] is a phase III trial examining the effects of finerenone in nondiabetic CKD, utilizing a randomized, double-blind, placebo-controlled, parallel-group, and multicenter design [42].

There exists compelling evidence supporting the use of steroidal MRAs in HFrEF, leading to reduced morbidity and mortality in affected patients. Nonetheless, this notable efficacy is accompanied by an increased risk of hyperkalemia, necessitating meticulous monitoring. In contrast, the evidence for MRA use in HFpEF is currently less conclusive.

The ongoing FINEARTS-HF study (NCT04435626) is designed to assess the efficacy and safety of finerenone in patients with HF and left ventricular ejection fraction greater or equal to 40% (HFpEF with or without T2DM). Participants will be randomly assigned to receive either 20 mg finerenone (for those with eGFR ≤ 60 mL/min/1.73 m^2^), 40 mg finerenone (for those with eGFR > 60 mL/min/1.73 m^2^), or a placebo. The study aims to evaluate the impact of finerenone on CV death and HF events [43].

Both the FIDELIO-DKD and FIGARO-DKD trials had a relatively small number of patients who were concurrently taking SGLT-2 inhibitors. However, in a multivariate analysis of the FIDELIO-DKD trial, it was observed that patients receiving SGLT-2 inhibitors were less likely to experience hyperkalemia [44]. Similarly, a sub-analysis of the FIGARO-DKD trial revealed a notable reduction in the risk of new-onset HF, and an exploratory analysis suggested that this reduction may have been more pronounced in patients who were also taking SGLT-2 inhibitors. Given the crucial role of SGLT-2 inhibitors in guideline-directed medical therapy (GDMT) for HF and CKD management, it becomes imperative for future studies to further explore and address this important question.

The ongoing phase 2 MIRACLE study (ClinicalTrials.gov identifier: NCT04595370) is evaluating the efficacy, safety, and tolerability of the selective MR modulator AZD9977 along with the SGLT-2 inhibitor dapagliflozin in patients with HF (LVEF < 60%) and CKD. Participants with HF and CKD (eGFR ≥ 20 to ≤60 mL/min/1.73 m^2^) will be treated with different doses of AZD9977 in combination with dapagliflozin (10 mg) or dapagliflozin (10 mg) alone [45].

The CONFIDENCE trial (ClinicalTrials.gov Identifier: NCT05254002) is a parallel-group treatment, phase II, double-blind, and three-arms study designed to investigate the combination effect of finerenone and empagliflozin in participants with CKD and T2DM using a UACR endpoint. The study will compare the effect of the combination of finerenone and empagliflozin with either drug administered alone on the relative changes from baseline in UACR at 180 days [46]. Since it is already established that a reduction in UACR is associated with more CV benefit and reduced CKD progression, if CONFIDENCE shows that the dual therapy with finerenone and SGLT2i can be used safely and has additive effects, it can be used for more effective CKD progression prevention [47].

The efficacy and safety of finerenone in CKD and T2DM with an eGFR below 25 mL/min/1.73 m^2^ remain unclear. A recent study [48] showed a significantly slower decline in eGFR in patients with CKD and T2DM with eGFR below 25. However, several limitations exist in this study including being non-randomized, retrospective, and single-center, and only nine patients are included in this study. Additionally, a large, randomized, and multicenter study is needed before reaching a definitive conclusion.

Other nonsteroidal MRAs like esaxerenone (CS-3150) have been shown to reduce albuminuria to normal levels if added to the regimen in patients already receiving therapy with RAAS inhibition drugs [49].

## 6. Conclusions

In conclusion, nonsteroidal mineralocorticoid receptor antagonists (MRAs) represent a promising advancement in the management of cardiorenal diseases. Finerenone, the first FDA-approved nonsteroidal MRA, has shown efficacy in HF and CKD, with significant improvements in kidney and CV outcomes. Ongoing trials, such as FINEARTS-HF, MIRACLE, and CONFIDENCE, explore further treatment possibilities. SGLT-2 inhibitors may potentially complement the benefits of finerenone, warranting future investigations. These groundbreaking agents hold potential as critical therapeutic options in the cardiorenal disease spectrum, addressing the limitations of steroidal MRAs and improving patient outcomes in this complex and challenging patient population.
